# Immunotherapy in the treatment of chemoresistant gestational trophoblastic neoplasia - systematic review with a presentation of the first 4 Brazilian cases

**DOI:** 10.1016/j.clinsp.2023.100260

**Published:** 2023-07-29

**Authors:** Antonio Braga, Elaine Balthar, Laís Cristhine Santos Souza, Michelle Samora, Matheus Rech, José Mauro Madi, Joffre Amim Junior, Jorge Rezende Filho, Kevin M. Elias, Neil S. Horowitz, Sue Yazaki Sun, Ross S. Berkowitz

**Affiliations:** a Rio de Janeiro Trophoblastic Disease Center, Maternidade Escola da Universidade Federal do Rio de Janeiro, RJ, Rio de Janeiro, Brazil; b Hospital Universitário Antonio Pedro da Universidade Federal Fluminense, RJ, Niterói, Brazil; c Postgraduate Program in Perinatal Health, Faculdade de Medicina, Maternidade Escola da, Universidade Federal do Rio de Janeiro, RJ, Rio de Janeiro, Brazil; d Postgraduate Program in Medical Sciences, Universidade Federal Fluminense, RJ, Niterói, Brazil; e Postgraduate Program in Applied Health Sciences, Universidade de Vassouras, RJ, Rio de Janeiro, Brazil; f Young Leadership Physicians Program, Academia Nacional de Medicina, RJ, Rio de Janeiro, Brazil; g Departament of Obstetrics, Escola Paulista de Medicina, Universidade Federal de São Paulo, SP, São Paulo, Brazil; h Caxias do Sul Trophoblastic Disease Center, Faculdade de Medicina, Universidade de Caxias do Sul (UCS), RS, Caxias do Sul, Brazil; i New England Trophoblastic Disease Center, Division of Gynecologic Oncology, Department of Obstetrics, Gynecology and Reproductive Biology, Brigham and Women's Hospital, Dana Farber Cancer Institute, Harvard Medical School, Boston, USA

**Keywords:** Gestational trophoblastic neoplasia, Immunotherapy, PD-1/PD-L1 inhibitors, Pembrolizumab, Avelumab

## Abstract

**Objective:**

To evaluate the efficacy of immunotherapy for GTN treatment after methotrexate-resistance or in cases of multiresistant disease, through a systematic review, as well as to present the first 4 Brazilian cases of immunotherapy for GTN treatment.

**Methods:**

Three independent researchers searched five electronic databases (EMBASE, LILACS, Medline, CENTRAL and Web of Science), for relevant articles up to February/2023 (PROSPERO CRD42023401453). The quality assessment was performed using the Newcastle Ottawa scale for case series and case reports. The primary outcome of this study was the occurrence of complete remission. The presentation of the case reports was approved by the Institutional Review Board.

**Results:**

Of the 4 cases presented, the first was a low-risk GTN with methotrexate resistance unsuccessfully treated with avelumab, which achieved remission with sequential multiagent chemotherapy. The remaining 3 cases were high-risk multiagent-resistant GTN that were successfully treated with pembrolizumab, among which there were two subsequent gestations, one of them with normal pregnancy and healthy conceptus. Regarding the systematic review, 12 studies were included, only one of them on avelumab, showing a 46.7% complete remission rate. The remaining 11 studies were on pembrolizumab, showing an 86.7% complete remission rate, regardless of tumor histology. Both immunotherapies showed good tolerability, with two healthy pregnancies being recorded: one after avelumb and another after pembrolizumab.

**Conclusion:**

Immunotherapy showed effectiveness for GTN treatment and may be especially useful in cases of high-risk disease, where pembrolizumab achieves a high therapeutic response, regardless of the histological type, and despite prior chemoresistance to multiple lines of treatment.

## Highlights

• Gestational trophoblastic neoplasia responds to anti-PD-1 or anti-PD-L1 immunotherapy.
• Multi-drug resistant gestational trophoblastic neoplasia may achieve remission with pembrolizumab.

## Introduction

Annually about 20,000 new cases of Gestational Trophoblastic Neoplasia (GTN) are diagnosed in the world.^1^^,^^2^ Although most of these cases originate from hydatidiform mole, GTN can develop after miscarriage, ectopic pregnancy, or delivery.^1^ Although GTN is largely cured with chemotherapy, multidrug-resistant cases can lead to death.^2^ Efforts have been made to establish novel treatments for these cases, as well as to lessen the immediate and cumulative side effects of the drugs used to treat GTN.^3^

The unique gestational nature of these tumors produces a high volume of paternally derived placental antigens which combined with the natural immunosurveillance of the fetal-maternal interface, results in a favorable environment for the use of immunotherapy in the treatment of these tumors.^4^

Of the most promising current immunotherapeutic targets in oncology are the drugs directed against programmed cell Death Protein 1 (PD-1) and its Ligands (PD-L1/2). Binding PD-L1/2 to its PD-1 transmembrane receptor normally inhibits effector T-cell activation, facilitating tumor-immune evasion. Drugs that inhibit this immune tolerance mechanism have been successfully used in the treatment of several solid tumors.^4^ As trophoblasts ubiquitously express PD-L1, blocking this pathway has been an area of key clinical interest.^5^

Pembrolizumab (which targets PD-1 on T-cells) was the first immunotherapy used for multi-drug resistant GTN, with a 75% (3/4 patients) Complete Response (CR).^6^ Subsequently, avelumab (inhibiting PD-L1 on trophoblasts) did not show good response in the treatment of multi-drug resistant GTN,^7^ although it achieved CR in 53.3% (8/15 patients) as first-line treatment after chemoresistance to Methotrexate (MTX).^8^ These results led the National Comprehensive Cancer Network to recommend immunotherapy as a therapeutic option for cases of chemoresistant GTN.^9^

The objective of this article is to evaluate the efficacy of immunotherapy in the treatment of GTN after MTX failure or in cases of multidrug-resistant disease, through a systematic review of the literature, as well as to present the first 4 Brazilian cases of immunotherapy for the treatment of GTN, two of whom achieved a subsequent pregnancy.

## Materials and methods

### Design

This systematic review was conducted according to the Cochrane Handbook for Systematic Reviews ^10^ and reported following the Preferred Reporting Items for Systematic Review and Meta-Analysis Protocols (PRISMA) recommendations.^11^ This study was registered at PROSPERO (March 7^th^, 2023), the International Prospective Register of Systematic Reviews, at the University of York (CRD42023401453).^12^

Additionally, 4 Brazilian cases of GTN treated with immunotherapy will be reported.

### Eligibility criteria for included studies

The authors included any case report, observational or interventional studies that evaluated the outcomes of immunotherapy treatment for GTN including avelumab after MTX failure or pembrolizumab after multiresistant disease. Animal studies, narrative reviews, case reports and expert opinions were excluded. GTN cases treated with other immunotherapy drugs were excluded. In particular, the authors did not include a reported Phase 2 trial evaluating camrelizumab plus apatinib, as responses to single-agent camrelizumab were not reported, and this agent is not widely available outside China.^13^

For systematic review the authors included studies that evaluated the GTN outcome to treatment with avelumab or pembrolizumab, that answer the question: *What is the complete response rate after immunotherapy in the treatment of GTN?* The PECO acronym was used, which corresponds to the areas P (Population), E (Exposition), C (Comparison) and O (Outcome):

1. Population: Women who had chemoresistant GTN (MTX-failure or multiresistant disease);
2. Exposition: Immunotherapy (avelumab or pembrolizumab);
3. Comparison: GTN treated with immunotherapy with resistance or toxicity that prevented the continuation of standard chemotherapy treatment;
4. Outcome: Complete remission after immunotherapy.

The detailed search strategy for each database is summarized in Supplemental Table 1 and additional search strategies can be accessed at PROSPERO.^12^ There was no publication year restriction.

### Search methods for identifying studies

The following keywords and Medical Subject Headings related to immunotherapy (avelumab or pembrolizumab) and GTN were used alone or in combination (and with synonyms and closely related words) to retrieve relevant articles: ((“Gestational Trophoblastic Disease” [All Fields]) OR (“choriocarcinoma” [All Fields])) AND (“Immunotherapy” [MeSH Terms]) AND ((“remission” [All fields]) OR (“persistent” [All fields]) OR (“progression”)) AND ((“chemoresistant”) OR (“refractory” [All fields]) OR (“resistant”[All fields]) OR (”non-respondent” [All fields])).

The authors searched in Excerpta Medica Database (EMBASE) (www.embase.com), Latin American and Caribbean Center on Health Sciences Information (LILACS) (https://lilacs.bvsalud.org/), Medline (https://pubmed.ncbi.nlm.nih.gov/), Cochrane Central Register of Controlled Trials (CENTRAL) (https://www.cochranelibrary.com/central) and Web of Science (www.webofscience.com). The authors did not restrain the search to a specific time period, including all registered references up to February 2023.

### Data collection

Three independent researchers (AB, MR and JMM) evaluated all titles and abstracts for the initial screening of the studies. A fourth author adjudicated any discrepancy (SYS). All selected articles were read in full to assess the eligibility of the studies according to described inclusion and exclusion criteria to be considered in the systematic review. The researchers extracted all data from the retrieved articles, independently, using a standardized data extraction sheet.

In case of duplicate publications and more than one publication of a preliminary study, we attempted to maximize the use of information by simultaneous evaluation of all available data but did not include the same group more than once as patients in the analysis.

The following information was extracted for each study (when available):

1. Study characteristics: title, author, country, design, language of publication, year of publication, sample size, number of centers;
2. Population characteristics: total number and number in comparison groups, age;
3. Exposition: Immunotherapy with avelumab or pembrolizumab;
4. Treatment with avelumab after MTX failure;
5. Treatment with pembrolizumab after multi-drug resistant disease (at least two multiagent sequential regimens);
6. Control: GTN treated with immunotherapy with resistance or toxicity that prevented the continuation of treatment;
7. Outcomes: Complete remission after immunotherapy.

### Quality and evidence assessment

The quality assessment was performed using the Newcastle Ottawa scale for case series and case reports that can be categorized into four domains: selection, ascertainment, causality, and reporting.^14^ These four domains with leading explanatory questions are summarized in Supplemental Fig. 1. Two independent researchers assessed the quality and the evidence (AB and JMM), independently, and a third author adjudicated any discrepancy (SYS).

### Diagnosis and treatment of gestational trophoblastic neoplasia

The authors adopted the International Federation of Gynaecology and Obstetrics (FIGO) 2000 diagnostic criteria for GTN: four or more plateaued human Chorionic Gonadotropin (hCG) levels over three weeks, or an increase of hCG levels for three or more consecutive measurements for at least two weeks, during postmolar follow up; a histopathologic diagnosis of choriocarcinoma or when hCG levels remain elevated, even if they are falling, 6-months or more from the evacuation of a molar pregnancy.^15^ However, as of 2018, patients with persistent but decreasing levels of hCG at 6 months post-evacuation were no longer treated, also according to the updated FIGO guidelines.^16^

The World Health Organization (WHO) scoring system based on risk factors (Supplemental Table 2) was used to decide the chemotherapy treatment.^1^,^3^ Patients diagnosed with WHO/FIGO risk score ≤6 (low-risk GTN) were treated with single-agent chemotherapy (preferably an MTX regimen followed by Actinomycin-D – Act-D or carboplatin, in cases of chemoresistance). Patients diagnosed with FIGO risk score ≥7 (high-risk GTN) received multiagent-chemotherapy (preferably etoposide, MTX, Act-D, cyclophosphamide, oncovin – EMA/CO, followed by etoposide, cisplatin, MTX, Act-D – EP/EMA or paclitaxel/cisplatin-paclitaxel/etoposide – TP/TE regimen in cases of chemoresistance).^1^^,^^3^

Patients with MTX chemoresistance who were willing and able to be treated with avelumab (anti-PD-L1 human monoclonal antibody, 10 mg/kg intravenously every 2 weeks) received this treatment. On the other hand, patients with GTN with chemoresistance to at least 2 sequential multiagent regimens were treated with pembrolizumab (anti-PD-1 human monoclonal antibody, initially 3 mg/kg or more recently 200 mg fixed dose, intravenously every 3 weeks) if they wished and this drug was available.

In both scenarios, immunotherapy was continued until disease remission, followed by 3‒5 cycles of consolidation or when resistance was diagnosed: increase in hCG levels or plateau (less than 10% drop) in 3 out of 4 consecutive weekly assessments, plus unacceptable toxicity and/or death. Likewise, side effects of immunotherapy were monitored according to Common Terminology Criteria for Adverse Events, Version 5.0, 2017 (CTCAE, 2017).^17^

#### Outcome

The primary outcome of this study was the occurrence of complete remission attested by 3 weekly hCG levels <5 IU/L.

### Ethics

The presentation of the case reports was approved by the Institutional Review Board of Maternidade Escola da Rio de Janeiro Federal University (cases 1‒3, CAAE: 62951522.0.0000.5275) and Escola Paulista de Medicina of Universidade Federal de São Paulo (case 4, CAAE: 60867522.4.0000.5505), according to the recommendations of the Brazilian National Research Ethics Committee (CONEP resolution 466/2012). All patients provided informed consent authorizing the publication of their treatment and anonymizing the cases. It should also be noted that all the recommendations provided for by the Helsinki Declaration regarding research involving human subjects were completely followed.

## Results

Brief description of Brazilian gestational trophoblastic disease reference center and presentation of case reports

Once diagnosed with Gestational Trophoblastic Disease (GTD), patients are referred from the public regulation system to the Reference Center (RC), as agreed by the line of care for women with GTD, established by the Brazilian Ministry of Health.^18^ Additionally, patients can obtain care at RC directly, without the need for an official referral, which also applies to those coming from the private or supplementary health system, since the RC work with an open door to care for everyone with GTD.^19^ Currently, Brazil has 47 GTD-RC throughout the country, all with the same minimal functioning criteria, among which included the presence of 1 medical oncologist, 1 obstetrician-gynecologist, 1 pathologist, 1 nurse and 1 social worker, all with a special interest in GTD.

### Case 1

A 26 year old primigravida presented with a molar pregnancy in 2018, with the development of GTN (FIGO I:6), and was treated with 11 cycles of 8-day MTX and Folinic Acid rescue (FA) with chemoresistance. She then underwent a hysterectomy outside the Gestational Trophoblastic Disease Reference Center (GTD-RC) and was diagnosed with Choriocarcinoma (CC). The patient did not receive adjuvant chemotherapy and hCG levels remained elevated after the surgery, and she was then referred to the GTD-RC. Fearing the side effects of intravenous chemotherapy, the patient requested immunotherapy with avelumab. After 9 cycles of this treatment, no toxic effects of immunotherapy were reported. However, hCG levels rose by 25% consecutively among 8^th^ and 9^th^ cycles (reaching a hCG level of 104 IU/L), which the authors considered resistance and administered the EMA/CO regimen. The patient achieved remission after 2 cycles of EMA/CO and received a further 3 cycles of consolidation chemotherapy. She remains in remission after 18 months.

### Case 2

Primigravida, 26 years old, presented with a molar pregnancy in 2018, with the development of GTN (FIGO I:5), successfully treated with 5 cycles of 8-day MTX/FA, followed by 3 consolidation cycles. After 10 months of remission, she presented with vaginal hemorrhage and elevated hCG levels (1,200 IU/L). Screening for metastases showed 2 pulmonary nodules measuring 1.5 and 2 centimeters (cm), in addition to a 3.5 cm hypervascular myometrial lesion. Treatment for GTN relapse was the EMA/CO regimen (8 cycles), followed TP/TE (4 cycles), with no sustained response. In these treatments, the patient had multiple episodes of grade III febrile neutropenia,^17^ even with the use of prophylactic Granulocyte Colony-Stimulating Factor (G-CSF). After TP/TE regimen failure, further screening showed an increase in the size and number of lung metastases (total of 4 metastases larger than 2 cm), with an hCG level of 17,000 IU/L. Given this scenario, pembrolizumab (200 mg fixed dose) was started, inducing remission after 3 cycles, followed by 3 more consolidation cycles, while monitoring potential toxicity during immunotherapy. No toxic effects of immunotherapy were reported. The patient was in remission for 22 months, when she became pregnant, with normal gestation, uneventful spontaneous vaginal delivery (with 37 weeks of gestation), healthy newborn (Apgar 8/9, weighing 2900g), and normal placental histopathology.

### Case 3

Primigravida, 29 years old was diagnosed with GTN (FIGO 1:2) after complete hydatiform mole in 2018. She was initially treated outside the GTD-RC using MTX and Act-D, developing resistance to both regimens. She was then referred to a reference center, where she received 6 cycles of the EMA-CO regimen (+3 consolidation cycles), achieving hCG normalization. Six weeks later, her hCG increased and she underwent a hysterectomy (with a histopathologic exam revealing an invasive mole) and 2 cycles of TP/TE regimen (+3 cycles of consolidation). Although hCG normalization had been reached again, the patient presented with hCG re-elevation after 15 weeks and a pulmonary lesion measuring 1.8 cm was detected on PET-CT. At this point, considering the difficulty in performing pulmonary nodulectomy due to hospital restrictions imposed by COVID-19 and the toxicity of sequential multidrug chemotherapy, the authors decided to initiate pembrolizumab (2 mg/kg). After 3 cycles she achieved remission and received 5 more cycles of consolidation, without toxicity. She remains in remission after 24 months.

### Case 4

Gravidity III parity II, 41 years old, had GTN after a spontaneous abortion (FIGO III:8) in 2019 and was sequentially treated with EMA/CO (8 cycles), TP/TE (5 cycles), EMA/EP (7 cycles) and Ifosfamide, Carboplatin and Etoposide (ICE) (4 cycles), showing chemoresistance to all of these regimens. The patient had disease progression with the development of a single 3 cm liver metastasis and a 2 cm vaginal lesion. In view of the extensive exposure to etoposide (>4g), the authors decided not to adopt the escalated EP regimen and start pembrolizumab (200 mg fixed dose), which achieved remission after 9 cycles, followed by 3 consolidation cycles. No toxic effects of immunotherapy were reported. The patient was in remission for 13 months, when she became pregnant, with a complete hydatidiform mole which was evacuated at 8 weeks of gestation. The patient is still in post-molar follow-up, with hCG of 560 IU/L, and falling.

### Systematic review

**Fig. 1:**
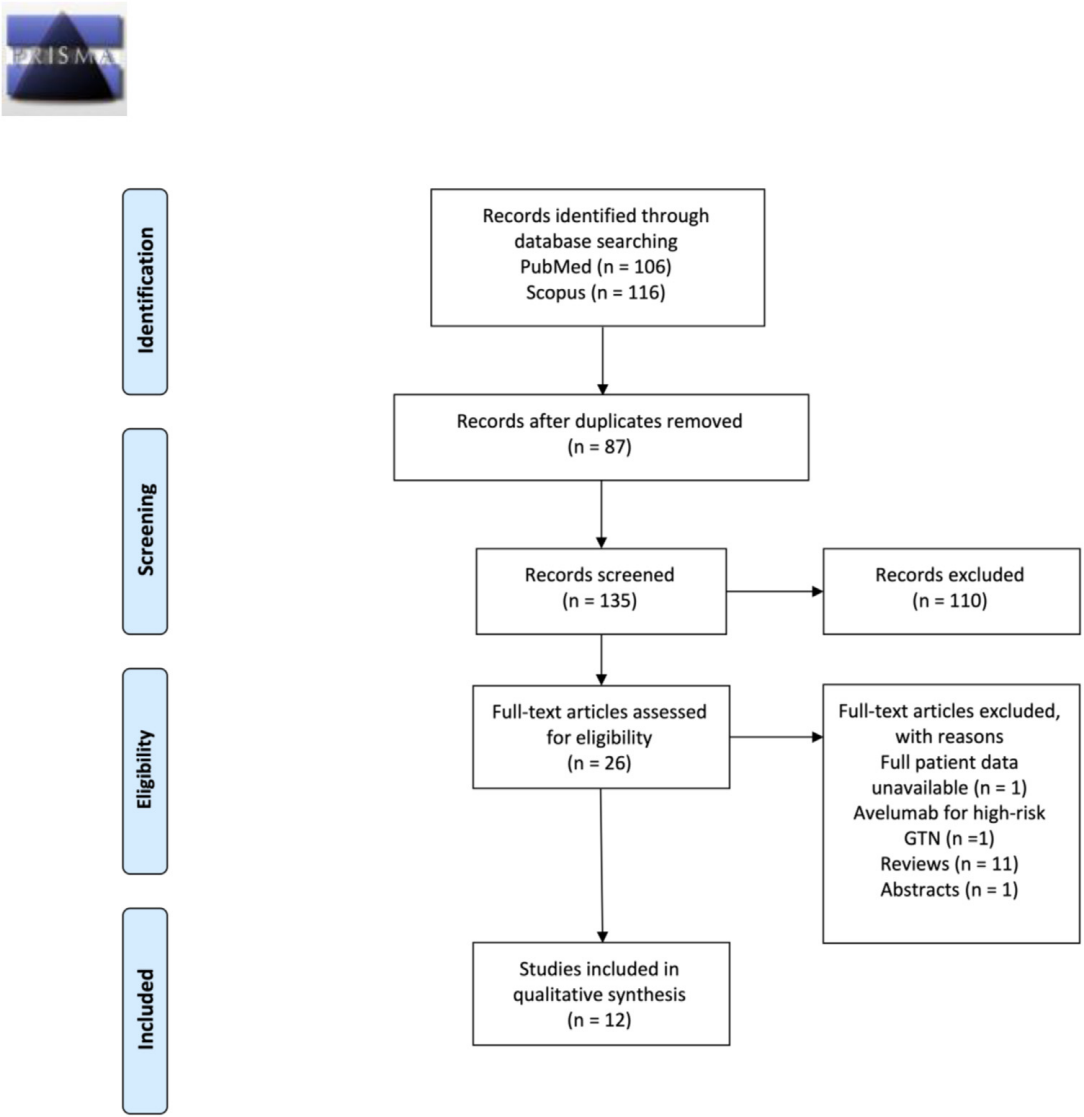
Prisma 2009 flow diagram.

A total of 134 studies on avelumab/pembrolizumab treatment for GTN were identified in the initial review. After initial screening, 25 potential full-texts were selected, among which 12 original studies were included in the systematic review and in the meta-analysis, as shown in Fig. 1.^5^^,^^7^^,^^20^, ^21^, ^22^, ^23^, ^24^, ^25^, ^26^, ^27^, ^28^, ^29^

The main characteristics of the included studies were summarized in Table 1. Six of the 12 studies were conducted in North America,^20^^,^^22^^,^^23^^,^^25^^,^^27^^,^^29^ four were conducted in Europe,^6^^,^^8^^,^^24^^,^^28^ one in Asia,^21^ and one between countries in North America and Europe.^26^

**Table 1:** Characteristics of the studies presenting results of avelumb and pembrolizumab for GTN treatment included in this systematic review.

Immunotherapy	Authors	Year	Type	Journal	Country	Number of case(s)
*Avelumab*	You et al.	2020	Clinical trial	Journal of Clinical Oncology	France	15
*Pembrolizumab*	Ghorani et al.	2017	Case series / Correspondence	Lancet	UK	4
	Huang et al.	2017	Case report / Correspondence	Journal of Clinical Oncology	US	1
	Choi et al.	2019	Case report	European Journal of Cancer	South Korea	2
	Clair et al.	2020	Case report	Gynecologic Oncology Reports	US	1
	Goldfarb et al.	2020	Case report / Correspondence	Gynecologic Oncology Reports	US	1
	Pisani et al.	2021	Case report	Current Oncology	Malta	1
	Bell et al.	2021	Case report	Gynecologic Oncology Reports	US	1
	Porter et al.	2021	Case report	Gynecologic Oncology Reports	US/UK	1
	Polnaszek et al.	2021	Case report	Obstetrics & Gynecology	US	1
	Paspalj et al.	2022	Case report	Gynecologic Oncology Reports	Austria	1
	Wong et al.	2022	Case report	Gynecologic Oncology Reports	US	1

There was only one study reporting the effect of avelumab in a clinical trial with 15 patients.^8^ They had GTN after a complete hydatidiform mole, a median age of 34 years, and developed resistance to single-agent chemotherapy (93% ‒ 14/15 treated with MTX and 7% ‒ 1/15 treated with Act-D) and were treated sequentially with avelumab. After a median of 9 cycles of immunotherapy, there was remission in 53.3% of patients (8/15), with no case of relapse after a median of 29 months of follow-up. Patients with avelumab resistance achieved remission with subsequent chemotherapy: three women (42.3%) were treated with actinomycin-D, 3 (42.3%) received multiagent chemotherapy, and 1 (14.3%) underwent hysterectomy. Avelumab was well tolerated, and no patient discontinued treatment due to toxicity. Only grade 1 and 2 adverse events were observed: fatigue, nausea, vomiting, infusion-related reaction, and diarrhea.

Regarding pembrolizumab, 11 studies were found with 15 reported cases,^6^^,^^20^, ^21^, ^22^, ^23^, ^24^, ^25^, ^26^, ^27^, ^28^, ^29^ summarized in Table 2. The median age of GTN patients treated with pembrolizumab was 39 years (1^st^/3^rd^ quartiles of 30.9 and 45.5 years, respectively). The most common histology of treated cases was CC (7/15 ‒ 46.7%), with 26.7% (4/15) of Placental Site Trophoblastic Tumor (PSTT), 20% (3/15) of Epithelioid Trophoblastic Tumor (ETT) and one case of mixed PSTT/ETT 6.6% (1/15).

**Table 2:** Clinical and oncologic outcomes of GTN patients treated with avelumab or pembrolizumab for GTN included in this systematic review.

Immunotherapy	Authors	Age	Histology	Number of cycles to remission	Number of consolidation cycles	Toxicity of (CTCAE grade)	Oncologic Outcome	Relapse after immunotherapy#	Reproductive outcomes
*Avelumab*	You et al.^a^	34^b^	All GTN cases are post CHM	8 (median) range 2‒11	3 (per protocol)	d	Remission in 8/15 (53.3%)	No cases reported (29 months)	e
*Pembrolizumab*	Ghorani et al.	39	CC	4	5	Arthralgia (G1)	Remission	No relpase after 24 months	Not reported
		44	Mixed PSTTand ETT	5	‒	Pruritis (G1)	Death	‒	TAH
		47	PSTT	8	5	Synovitis (G2) Rash (G1)	Remission	No relpase after 15 months	Not reported
		37	CC	2	5	Neutropaenia (G2) Synovitis (G1)	Remission	No relpase after 5 months	Not reported
	Huang et al.	26	CC	2	2	Hepatotoxicity (G3)	Remission	No relpase after 2 months	Not reported
	Choi et al.	39	PSTT	1	13	Not reported	Remission	No relpase after 29 months	TAH
		26	ETT	11	4**	Rash (G2)	Remission	***	TAH
	Clair et al.	30	CC	10	Not reported	Not reported	Remission	No relpase after 31 months	TAH
	Goldfarb et al.	50	CC	3	3	Peripheral neuropathy (G3)	Progression^1^	Under treatment	TAH
	Pisani et al.	49	ETT	Not reported	Not reported	Not reported	Remission	No relpase after 12 months	TAH
	Bell et al.	47	ETT	29 cycles ***	‒	Not reported	Remission	***	Not reported
	Porter et al.	34	PSTT	3	Not reported^2^	Inflammatory thyroiditis (no grade reported)	Remission	***	TAH
	Polnaszek et al.	23	PSTT	3	‒	Not reported	Remission	No relpase after 12 months	3
	Paspalj et al.	31	CC	4	3	Not reported	Remission	No relpase after 24 months	TAH
	Wong et al.	44	CC	****	****	Arthralgia (G1)	Remission	Relpase after 6 months*****	Not reported

^c^Complete hydatidiform mole.

* Need to reduce the dosage of the 2 consolidation cycles of pembrolizumab in 50% due to toxicity.

** The institution's tumor board decided to continue treatment with pembrolizumab, even after remission.

*** The patient was still undergoing consolidation chemotherapy at the time of publication of the case report.

**** The patient achieves remission after 2 cycles of pembrolizumab (followed by 5 consolidation cycles). However, she relapsed after 6 months and was again treated with pembrolizumab. The report was unclear but suggested that the patient achieved remission after 4 further cycles of pembrolizumab, followed by 21 consolidation cycles.

***** After GTN relapse notwithstanding the treatment with pembrolizumab, the patient was rescued with pembrolizumab and achieved remission again, with no evidence of disease and with normal hCG levels after 24 months of the end of immunotherapy.

# Between parentheses, the follow-up time, in months, after remission is presented, using the median for the study by You et al. CTCAE, Common Terminology Criteria for Adverse Events (reference 16); CC, Choriocarcinoma; PSTT, Placental site trophoblastic tumor; ETT, Epithelioid trophoblastic tumor; TAH, Underwent Total Abdominal Hysterectomy before pembrolizumab.

a Clinical trial with 15 patients treated with avelumab.

b Median.

d Two cases hyperthyroidism (13.3%), one case of hypothyroidism (6.7%); and one case of a grade 2 ovarian cyst (6.7%) and another case of a grade 3 uterine bleeding (6.7%), which were both unrelated to treatment.

e One case of healthy baby born vaginally at 39 weeks of gestation.

1 . Relapse after 8 months. She was followed up with hCG monitoring and imaging exams until reinitiating pembrolizumab after 14 months from the relapse.

2 . Although the number of consolidation chemotherapy cycles with pembrolizumab was not reported, the authors reported that they used, in addition to pembrolizumab, 5 consolidation cycles with the EP/EMA regimen, replacing cisplatin to carboplatin in the last cycle due to toxicity (thrombocytopenia, ototoxicity and tinnitus). Finally, the authors reported that she is still on consolidation treatment with pembrolizumab.

3 . Since the diagnosis of PSTT, the patient has refused to undergo hysterectomy or even conventional chemotherapy for PSTT, accepting only treatment with pembrolizumab. The patient achieved remission with immunotherapy and became pregnant during consolidation chemotherapy with pembrolizumab, which was immediately discontinued. The pregnancy progressed uneventfully with a delivery of a healthy baby born vaginally at 39 weeks of gestation.

**Fig. 2:**
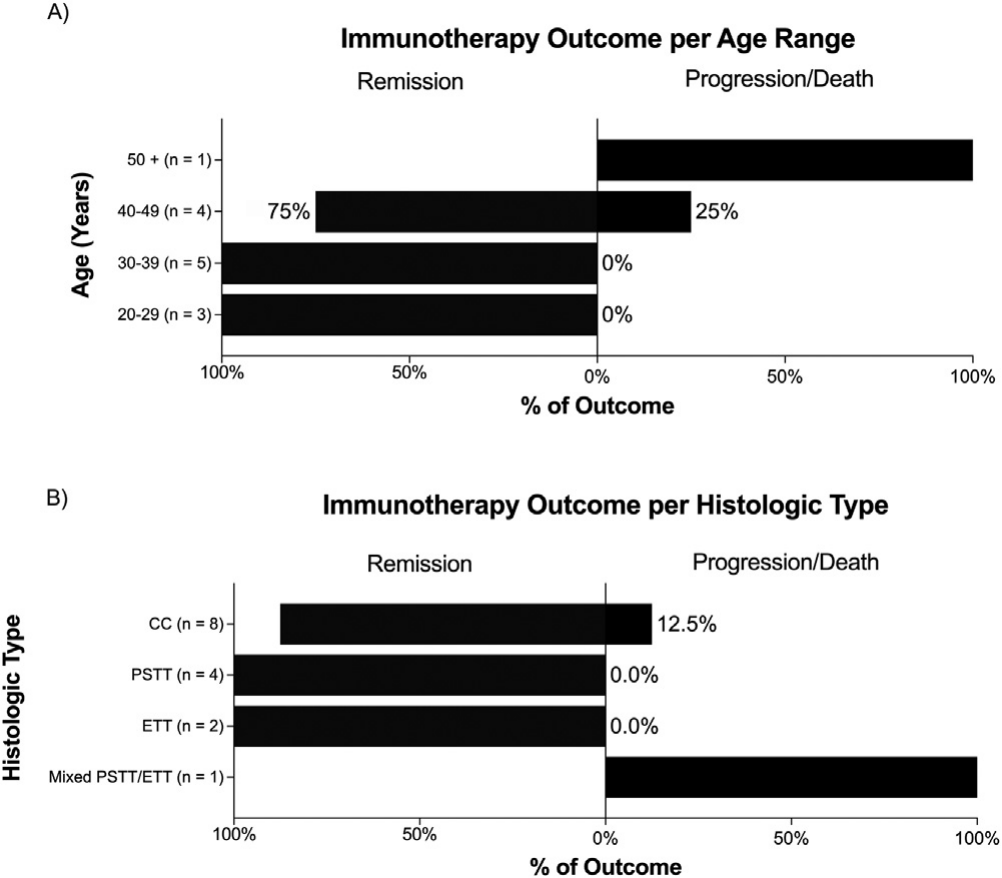
Immunotherapy outcomes for gestational trophoblastic neoplasia according to the patient's age and histologic tumor classification. CC, Choriocarcinoma; PSTT, Placental Site Trophoblastic Tumor; ETT, Epithelioid Trophoblastic Tumor.

Pembrolizumab induced remission in 86.7% (13/15) of GTN chemoresistant to multiagent regimens. Excluding the case of Bell et al. (the tumor board decided to maintain pembrolizumab even after remission and the publication of the article)[25] and the case of Wong et al. (who relapsed after remission achieved with pembrolizumab, was once more treated with this regimen and achieving remission over again),^29^ the other cases in which there was sustained remission received a median of 3.5 cycles of prembrolizumab to achieve remission (1^st^/3^rd^ quartiles of 2.75 and 5.75 cycles, respectively). A median of 4 cycles of consolidation chemotherapy with pembrolizumab was given after remission (1^st^/3^rd^ quartiles of 3 and 5 cycles, respectively). Fig. 2A shows that there was a 40% (2/5) failure of treatment with pembrolizumab among patients ≥ 40 years old, while it was associated with remission in all 10 patients ≤ 39 years old. Furthermore, Fig. 2B shows that pembrolizumab was effective in cases of CC, PSTT and ETT, with no response in the only case of mixed PSTT/ETT and in one case (12.5%) of CC.

Treatment with pembrolizumab was very well tolerated, with CTCAE[17] grade III toxicity occurring only in 13.3% (2/15) of patients, of which in one it was necessary to discontinue treatment due to peripheral neuropathy[23] and, in the other, a 50% reduction in the dose of pembrolizumab was sufficient to control hepatotoxicity.^20^

Although Total Abdominal Hysterectomy (TAH) was done in 8/15 patients prior to the treatment with pembrolizumab, one of them, who maintained the uterus, became pregnant. It was noteworthy that this pregnancy occurred during treatment with pembrolizumab, continuing without complications until the vaginal birth, in the 39^th^ week of gestation, of a healthy conceptus.^27^

## Discussion

This systematic review shows that immunotherapy, being administered alone or in combination with traditional cytotoxic chemotherapy, is a safe and effective option for GTN treatment.^4^^,^^6^^,^^8^^,^^20^, ^21^, ^22^, ^23^, ^24^, ^25^, ^26^, ^27^, ^28^, ^29^, ^30^ There is also new data evaluating PD-1 inhibitors being administered with traditional cytotoxic chemotherapy in GTN with encouraging results. Avelumab results are modest in the second-line GTN treatment after resistance to single-agent chemotherapy (53.3%), especially when considering the results of conventional chemotherapy for these cases (Act-D or carboplatin), whose remission rates are minimally equal or higher (50%‒80%),^31^, ^32^, ^33^, ^34^, ^35^ with a well-tolerable toxicity pattern and much lower costs. However, the outcomes of pembrolizumab for GTN resistant to several lines of multiagent chemotherapy are promising, achieving remission in 86.7% (13/15) of treated cases. This article also presents the first 4 cases of immunotherapy in the treatment of GTN in the Southern Hemisphere, reinforcing the excellent results of pembrolizumab, with 100% of remission after multiagent chemoresistant GTN and failure of avelumab for second-line low-risk GTN treatment after MTX resistance.

The systematic review showed that pembrolizumab is effective for the treatment of GTN regardless of its histological subtype, achieving a good response in cases of CC, PSTT, or ETT. This is important because it will allow patients without a histological diagnosis of GTN to be treated with pembrolizumab without the need for hysterectomy for histopathological evaluation of tumors confined to the uterus, or even to assess PDL-1 marker immunoexpression. The Brazilian cases reported here illustrate this assertion by showing that, in 2/3 of the cases treated with pembrolizumab, they occurred in young women or women with reproductive desire, for whom hysterectomy would prevent a new pregnancy. Although hysterectomy, followed or not by chemotherapy, is the treatment of choice for cases of PSTT or ETT, the report by Polnaszek et al. in which a patient with PSTT refused hysterectomy and was successfully treated with pembrolizumab, even with a new pregnancy,^27^ may be an option for exceptional cases. These challenging situations sometimes appear in GTN referral centers and fertility-sparing treatments for PSTT and ETT cases have been increasingly discussed in the literature.^36^

The systematic review also found that the response to pembrolizumab decreased with increasing age in patients with GTN, especially over 40‒50 years. This eventual immunological senescence, known as age-related immune dysfunction, has been much debated in treatments with immune checkpoint inhibitors.^37^^,^^38^ Although there seems to be a paradoxical better response to immunotherapy in those ≥65 years old, the results of the treatment of other solid tumors with pembrolizumab show more unfavorable results in patients ≤40 years, when compared with those older,^37^^,^^38^ unlike what the authors found with the GTN patients included in this systematic review. As GTN affects women of reproductive age, future research should take age difference into consideration in order to assess the real impact of age on the GTN response to immunotherapy, especially when the authors consider that, in cases of conventional chemotherapy, increasing age is associated with a worse prognosis.^16^

It is also worth mentioning that the authors presented, to the best of our knowledge, the third and fourth cases of gestations after immunotherapy for GTN, one of which had an uneventful pregnancy and a healthy conceptus born at term and the other case was a recurrent hydatidiform mole in a 44-year-old. When pregnancy occurs ≤6 months after the end of chemotherapy, there seems to be a greater chance of miscarriage, while in pregnancies that occur ≥ 12 months after the end of chemotherapy, the only risk maintained seems to be that of recurrent hydatidiform mole, especially in women over ≥ 40 years old.^39^, ^40^, ^41^ There are concerns about the fertility of menstruating women treated with immunotherapy,^42^ not only about the possible risk of infertility that may occur by an exacerbated immunological reaction to the ovarian follicles,^43^ as well as a possible rejection of the conceptus and fetal loss,^44^ directly induced or mediated by hypothyroidism, one of the most common adverse events of immunotherapy.^45^ Reports of pregnancies after immunotherapy, especially those with a successful outcome, such as the one presented in this article, are encouraging.

This systematic review included studies from 6 countries indicating that this may allow the results to be globally generalizable. The main limitation of this review is the rarity of GTN cases treated with immunotherapy, causing almost exclusively case reports to be included in this systematic review. The use of the Newcastle Ottawa scale for the quality assessment of the case series and case reports included in this review showed the specific weaknesses of these articles. Among these, the authors highlight the lack of some treatment details and the short follow-up time after remission that may have limited the diagnosis of GTN relapse. However, the involvement in this study of authors recognized as specialists in GTN may have facilitated a better interpretation of the results.

## Conclusions

Although avelumab has shown effectiveness as a second-line treatment for low-risk GTN with MTX (or even Act-D) chemoresistance, in developing countries, its high cost will bring difficulties for its implementation, especially when considering that the therapeutic options are equally safe and with similar or better performance. Pembrolizumab, on the other hand, appears to be an option with a high therapeutic response, regardless of the histological type, and despite prior chemoresistance to multiple lines of treatment.

## Authors’ contributions

AB, JMM, KME, NSH and RSB contributed to the conception of the study protocol and search strategy. The manuscript of the protocol was drafted by AB, MR and JMM, and was critically revised by KME, NSH and RSB. AB, MR, JMM, KME, NSH, SYS, and RSB analyzed the results and made the necessary clinical correlations. AB, EB, SYS, LCSS, MS, and GF treated the new cases reported. All authors wrote and approved the final version of the paper.

## Declaration of Competing Interest

The authors declare no conflicts of interest.

## Appendix Supplementary materials

Image, application 1

## Footnotes

Supplementary material associated with this article can be found in the online version at doi:10.1016/j.clinsp.2023.100260.
