# Supplementary material for: Immunotherapy in the treatment of chemoresistant gestational trophoblastic neoplasia - systematic review with a presentation of the first 4 Brazilian cases
Source: Clinics (Sao Paulo). 2023 Jul 29;78:100260. doi: 10.1016/j.clinsp.2023.100260 (PMC10404605; doi:10.1016/j.clinsp.2023.100260)

**CLINICS-D-23-00305_Supplementary Material**

**Supplemental Table 1** Detailed searching strategy on immunotherapy for gestational trophoblastic neoplasia.

| **Database** | **Search Strategy** | **Number of papers outputted; selected** |
| --- | --- | --- |
| PUBMED | (("Gestational Trophoblastic Disease"[All Fields]) OR "gestational trophoblastic neoplasia") AND (("Choriocarcinoma") OR (("placental site trophoblastic tumor") OR ("PSTT") OR ("epithelioid trophoblastic tumor") OR (“ETT”) [All terms])) AND (("Immunotherapy"[All fields]) OR "pembrolizumab"[All fields] OR "avelumab"[All fields]) AND (("PD-1/PDL-1") OR ("programmed cell death-1/ programmed cell death ligand-1") AND ((“remission”) OR (“progression”) OR (“death”)) | 106 |
| SCOPUS | TITLE-ABS-KEY((“Gestational Trophoblastic Disease” OR “gestational trophoblastic neoplasia”) AND (“Choriocarcinoma” OR “placental site trophoblastic tumor” OR “PSTT” OR “epithelioid trophoblastic tumor” OR “ETT”) AND (“Immunotherapy” OR “pembrolizumab” OR “avelumab”) AND (“PD-1” OR “PDL-1” OR “programmed cell death 1” OR “programed cell death ligand 1”) AND (“remission” OR “progression” OR “death”)) | 116 |

**Supplemental Table 2** World Health Organization/International Federation of Gynecology and Obstetrics (WHO/FIGO) staging and classification of gestational trophoblastic disease^a^.

| **GTN: FIGO staging and classification (Washington, 2000)** | | | | |
| --- | --- | --- | --- | --- |
| **FIGO anatomic staging:** | | | | |
| Stage I: Disease confined to the uterus | | | | |
| Stage II: GTN extends outside of the uterus, but is limited to the genital structures (adnexa, vagina, broad ligament) | | | | |
| Stage III: GTN extends to the lungs, with or without known genital tract involvement | | | | |
| Stage IV: All other metastatic sites | | | | |
| **Modified WHO prognostic scoring system as adapted by FIGO** | | | | |
| **Prognostic factors** | **Score** | | | |
|  | **0** | **1** | **2** | **4** |
| Age (years) | <40 | ≥40 | ‒ | ‒ |
| Antecedent gestation | mole | abortion | term | ‒ |
| Interval^b^ (months) | <4 | 4‒6 | 7‒12 | >12 |
| Pretreatment serum hCG (IU/L) | <10^3^ | 10^3^ to <10^4^ | 10^4^ to <10^5^ | >10^5^ |
| Largest tumor size (including uterus) | <3 | 3 to 4 | ≥5 | ‒ |
| Site of metastases | Lung | Spleen, kidney | Gastro intestinal tract | Brain, liver |
| Number of metastases | ‒ | 1‒4 | 5‒8 | >8 |
| Previous failed chemotherapy | ‒ | ‒ | single drug | 2 or more drugs |

^a^ We have received written permission to reproduce this Table by John Wiley and Sons Copyright License Number 5536450082000.

^b^ Interval (in months) between the end of antecedent gestation (when known) and the beginning of chemotherapy.

GTN, Gestational trophoblastic neoplasia; hCG (IU/L), Human Chorionic Gonadotropin (International Units per liter).

**Supplemental Figure 1** Quality assessment for case series and case reports using the Newcastle Ottawa scale.


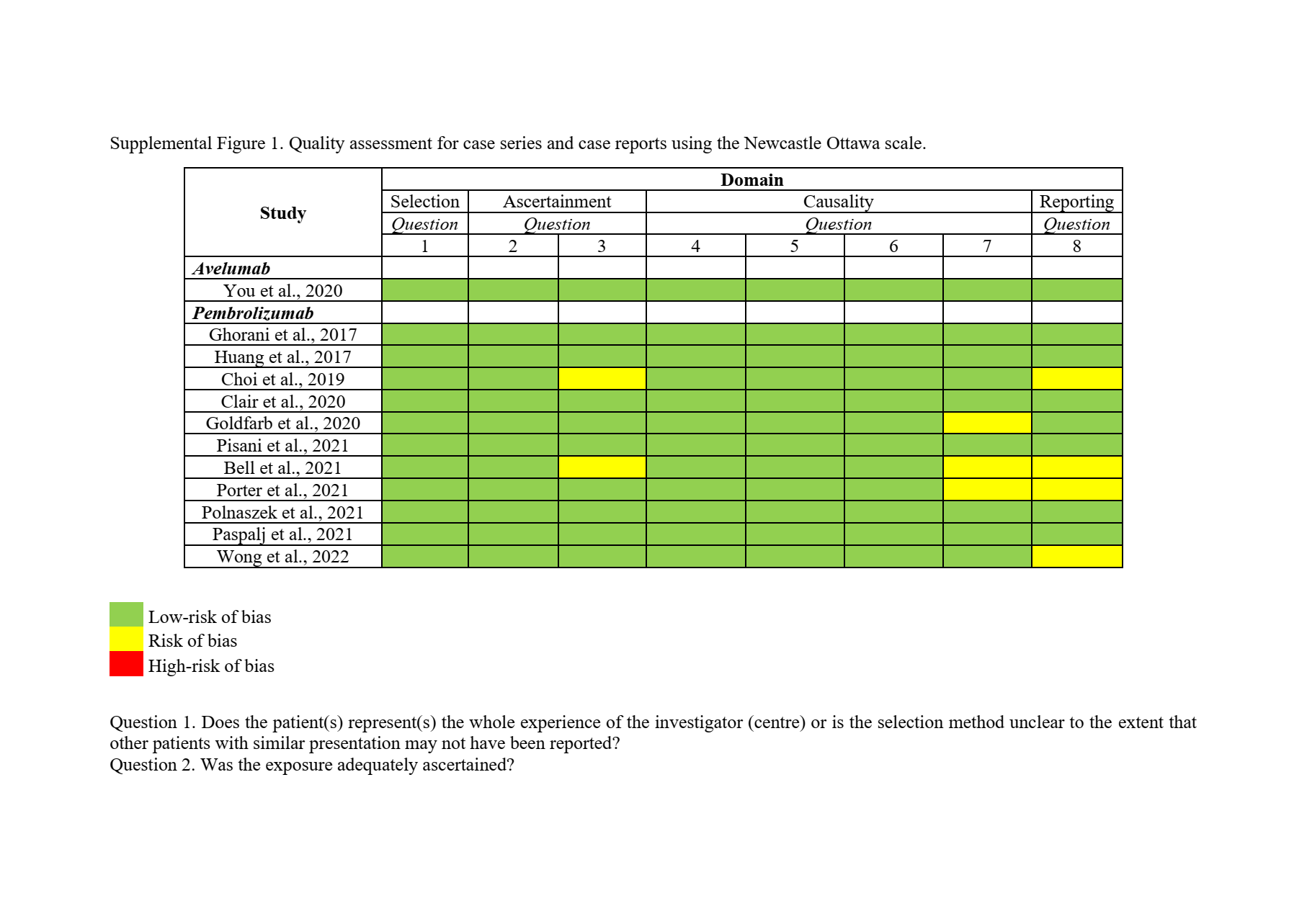

Supplement: Supplementary file 1 [file mmc1.doc]
